# Repository Corticotropin Injection for Treatment of Idiopathic Inflammatory Myopathies

**DOI:** 10.1155/2016/9068061

**Published:** 2016-08-23

**Authors:** Aarat Patel, Georgia Seely, Rohit Aggarwal

**Affiliations:** ^1^Arthritis and Osteoporosis Center of Richmond, 9600 Patterson Avenue, Richmond, VA 23229, USA; ^2^Department of Pediatrics, University of Virginia Children's Hospital, Box 800386, Charlottesville, VA 22908, USA; ^3^Dermatology Associates of Virginia, P.C., 10800 Midlothian Turnpike, Suite 309, Richmond, VA 23226, USA; ^4^Division of Rheumatology and Clinical Immunology, University of Pittsburgh, BST S725, 3500 Terrace Street, Pittsburgh, PA 15217, USA

## Abstract

Idiopathic inflammatory myopathies are a group of systemic autoimmune diseases that involve inflammation of skeletal muscle. The two most common forms are dermatomyositis and polymyositis, the former of which entails a skin component. There are few approved therapeutics available for treatment of this group of diseases and the first-line therapy is usually corticosteroid treatment. Considering that a large proportion of patients do not respond to or cannot tolerate corticosteroids, additional treatments are required. There are second-line therapies available, but many patients are also refractory to those options. H.P. Acthar® Gel (repository corticotropin injection [RCI]) is a melanocortin peptide that can induce steroid-dependent effects and steroid-independent effects. Herein, we present a series of cases that involved the use of RCI in the management of dermatomyositis and polymyositis. RCI treatments resulted in improvement in three of four patients, despite failure with previous therapies. The use of RCI did not exacerbate any comorbidity and no significant changes in blood pressure, weight, or glycemic control were observed. Overall, these results are encouraging and suggest that randomized, controlled clinical trials applying RCI to dermatomyositis and polymyositis are warranted.

## 1. Introduction

Idiopathic inflammatory myopathies (commonly referred to as myositis) describe a group of systemic autoimmune muscle diseases characterized by inflammation of skeletal muscle, the most common of which include dermatomyositis (DM) and polymyositis (PM) [1]. There are limited approved therapeutic agents used to treat DM/PM, but the first-line therapy is corticosteroid treatment [1, 2]. Thirty to forty percent of patients cannot tolerate or do not completely respond to high-dose corticosteroids, and up to 30% of patients do not respond to biologics [3, 4]. These patients require additional immunosuppressive agents, yet significant numbers of patients are refractory to these drugs. H.P. Acthar Gel (ACTH_1–39_, repository corticotropin injection [RCI]) is a highly purified analogue of ACTH in gelatin that provides prolonged release and is approved for use in DM/PM [5]. ACTH is a melanocortin peptide that can induce a steroid-dependent effect and a broader, steroid-independent, anti-inflammatory effect. Melanocortins exert their effects through engagement of the melanocortin receptors (MC_1_–MC_5_) [6]. Current literature regarding the use of RCI in patients with DM/PM is limited and this case series demonstrates the efficacy of RCI treatment in those that are refractory to first-line corticosteroid treatments and several secondary treatment options.

## 2. Case Reports

A 70-year-old Caucasian female presented with erythematous rashes, abnormal nail-fold capillary changes, and pruritus. There was no serological or clinical evidence of muscle involvement, but she had classic DM skin rashes of Gottron's papule. Skin biopsy led to clinical diagnosis of amyopathic dermatomyositis. She was treated with prednisone, topical corticosteroids, and systemic medications (Table 1); however, there was an incomplete response. The patient was then prescribed RCI (80 IU twice weekly SQ). During RCI treatment, pruritus and rash resolved; the violaceous erythema remained minimally active on the upper back. Upon sun exposure, the patient suffered a flare-up of the rash; however, this resolved without change in therapy. At the time of the last visit, the patient remained on RCI but had a moderate flare of rash; another rituximab infusion has been discussed. Bone density has improved since being on RCI.

A 50-year-old Caucasian male presented with an erythematous rash without myalgia or arthralgia despite mildly elevated lactate dehydrogenase (LD) and aldolase. The skin rashes were severe and distributed in a classic DM pattern with Gottron's papules, shawl sign, holster sign, v-neck pattern, and heliotrope rash. Electromyography (EMG) and skin biopsy were consistent with DM and malignancy was ruled out. The patient was prescribed prednisone and azathioprine (AZA). There was incomplete response with persistent rashes at 3 months, as well as weight gain related to corticosteroid use. RCI was prescribed (80 IU twice weekly SQ) and prednisone was tapered off without worsening of the rash. The dose of AZA was increased and other treatments, including intravenous immunoglobulin (IVIg) and methotrexate (MTX), were added to RCI due to active stable rash. The patient's rash persisted despite normalization of muscle enzyme levels (LD and aldolase) and all treatments, including RCI, were discontinued after 8 months (Table 2). The patient continued MTX and rituximab, hydroxychloroquine, and mycophenolate mofetil (MMF) were prescribed. Prednisone was reintroduced. During rituximab treatment, his rashes improved but did not completely resolve.

A 52-year-old Caucasian female presented with proximal muscle weakness in the lower extremities, puffy fingers, nail-fold capillary changes, myalgia, and fatigue. Muscle enzymes (CK, LD) were elevated (Table 2) and the patient was noted to have interstitial lung disease (ILD) and Sjögren's syndrome. A nonspecific interstitial pneumonia was noted on lung biopsy. Clinical diagnosis of PM was made based on clinical features and EMG. At the time of presentation, the patient was taking prednisone (5 mg a day) for ILD and was started on MMF for muscle weakness. During this treatment, the proximal muscle weakness and puffy fingers improved; however, a higher dose of prednisone (20 mg a day) was prescribed to control muscle symptoms and dyspnea from ILD. Muscle enzymes remained elevated, muscle strength did not improve, and the patient experienced insomnia, anxiety, and weight gain with higher doses of prednisone. MMF was increased (2000 mg a day) and RCI (80 IU twice weekly SQ) was added as a corticosteroid sparing agent (Table 1). The patient reported less fatigue upon starting RCI and had no adverse events (AEs) during treatment. The patient was able to taper prednisone during RCI treatments, which relieved steroid related side effects. Muscle enzymes, muscle strength, and myalgia improved with RCI treatment; however, there was no improvement in lung function. Rituximab was added in an effort to alleviate dyspnea from ILD, and lung function improved as a result. At the time of the last visit, the patient was still receiving MMF, RCI, and prednisone. Muscle enzyme levels and muscle strength continue to be normal.

A 57-year-old African American male presented with severe proximal muscle weakness, dysphagia, arthralgia, and fatigue. Muscles enzymes were elevated (Table 2) and anti-SRP-antibody was noted. Muscle biopsy was consistent with PM. The patient received pulse corticosteroids, oral prednisone, and IVIg (Table 1) with minimal improvements. The addition of AZA did not mitigate symptoms or decrease muscle enzymes. IVIg became ineffective after several months, so rituximab was given. This resulted in minimal improvement in muscle enzymes and weakness. At this point, prednisone was ineffective, so RCI (80 IU twice weekly SQ) was added to the treatment strategy. The patient reported improved proximal weakness after a few weeks on RCI and muscle enzymes were normalized. Muscle strength was found to have improved on examination. The patient was able to successfully wean off of prednisone while on RCI. The patient has been on RCI monotherapy for 27 months and has had no significant adverse effects.

## 3. Discussion

We assessed the efficacy of RCI in mediating DM/PM progression in four separate cases. All patients in this case series were refractory to corticosteroids, in addition to other disease-modifying agents, and were subsequently prescribed RCI. RCI is thought to play an immunomodulatory role through engagement of the melanocortin receptors in the immune system but may also activate receptors in skeletal muscle [7]. Most patients experienced improvement in clinical laboratory measures, muscle strength, and pain upon the addition of RCI, though skin response was variable.

Of note, RCI use did not exacerbate comorbidities in these patients. Case 2 had no exacerbation of ILD; however, RCI provided no improvement to the lung disease. There were no significant changes in blood pressure recorded in any patient. Hyperglycemia was not an issue while on RCI, even for the patient with diabetes (Case 3). There were no significant changes in weight observed in Cases 1, 2, and 4 during the RCI treatment period. Case 3 had mild weight gain; however, she was taking prednisone while on RCI. Case 1 reported blurry vision during RCI treatment; however, this resolved while on treatment. No changes in skin pigmentation were noted in these patients after addition of RCI to their treatment regimen. Evidence of improvement of bone density while on RCI without use of bisphosphonates was observed in Case 1. The limitations of our case series are the small sample size and the retrospective nature of our study; it is also difficult to understand the contribution of concomitant therapies to overall response.

## 4. Conclusion

RCI treatment resulted in variable improvement for three of four patients suffering from DM/PM, despite failing to completely respond to first-line corticosteroid treatments, as well as other immunosuppressive agents. Our results are similar to a recent retrospective case series of patients with refractory myositis (three DM, two PM) receiving RCI. All patients showed improvement in muscle strength, as well as resolution of rash, without major side effects [8]. These results are encouraging and larger randomized, controlled clinical trials are needed. Currently, there is a larger open label clinical trial underway to further evaluate efficacy and safety of RCI in PM and DM patients [9].

## Figures and Tables

**Table 1:** Patient age at diagnosis, therapy prior to RCI, medications concomitant with RCI, and comorbid conditions.

Case	Age/gender diagnosis	Prior failed therapies	Concomitant medications	Comorbid conditions
1	70/F Amyopathic DM	Prednisone (up to 60 mg/day) MMF (1000 mg bid) AZA (100 mg qd) CYC (100 mg bid) Etanercept (50 mg) Infliximab (5 mg/kg) IVIg (2 g/kg) Adalimumab (40 mg) Thalidomide (100 mg qd) MTX (15 mg) Hydroxychloroquine (200 mg bid) Quinacrine (100 mg) Ustekinumab (45 mg)	Rituximab (1000 mg) Prednisone (5 mg/day with tapering off)	

2	50/M DM	Prednisone (up to 60 mg/day) AZA (50 mg bid)	Prednisone (30 mg/day with tapering) AZA (200 mg/day) IVIg (2 g/kg) MTX (20 mg)	

3	52/F PM	Prednisone (up to 20 mg/day) MMF (1000 mg bid)	Prednisone (20 mg/day with tapering to 5 mg) MMF (2000 mg bid)	ILD Sjögren's syndrome Primary biliary cirrhosis Diabetes

4	57/M PM Anti-SRP antibody positive	IV steroids (1 g/day for 3 days) Prednisone (up to 60 mg/day) IVIg (2 g/kg) AZA (50 mg bid) Rituximab (1000 mg)	Prednisone (10 mg/day with tapering off)	Hepatitis C Hypertension Hyperlipidemia

MMF: mycophenolate mofetil; AZA: azathioprine; CYC: cyclosporine; MTX: methotrexate; ILD: interstitial lung disease.

**Table 2:** Clinical laboratory values for each case before and after treatment with RCI.

	Case 1		Case 2		Case 3		Case 4	
	Before RCI	After RCI	Before RCI	After RCI	Before RCI	After RCI	Before RCI	After RCI
Creatine kinase (total, 24–173 U/L)	48	72	143	51	1785	132	6753	403
Lactate dehydrogenase (119–226 U/L)	NA	NA	280	216	466	222	1605	204
Aspartate transaminase (0–40 U/L)	14	28	36	28	99	63	163	23
Alanine transaminase (0–32 U/L)	26	18	48	18	112	125	74	23
Aldolase (3.3–10.3 U/L)	NA	NA	11	6	22.1	8.8	48.8	5.7
Muscle weakness (mild, moderate, and severe)	None	None	None	None	Mild	None	Severe	None
Myalgia (mild, moderate, and severe)	None	None	Mild	Mild	Moderate	None	Severe	None
Prednisone dose (mg)	60 mg	None	30 mg	5 mg	20 mg	5 mg	10 mg	None
